# Supplementary material for: Adhesion toughness of multilayer graphene films
Source: Nat Commun. 2017 Dec 5;8:1952. doi: 10.1038/s41467-017-02115-w (PMC5717257; doi:10.1038/s41467-017-02115-w)
Supplement: Supplementary file 1 — Supplementary Information [file 41467_2017_2115_MOESM1_ESM.pdf]

Supplementary Figures

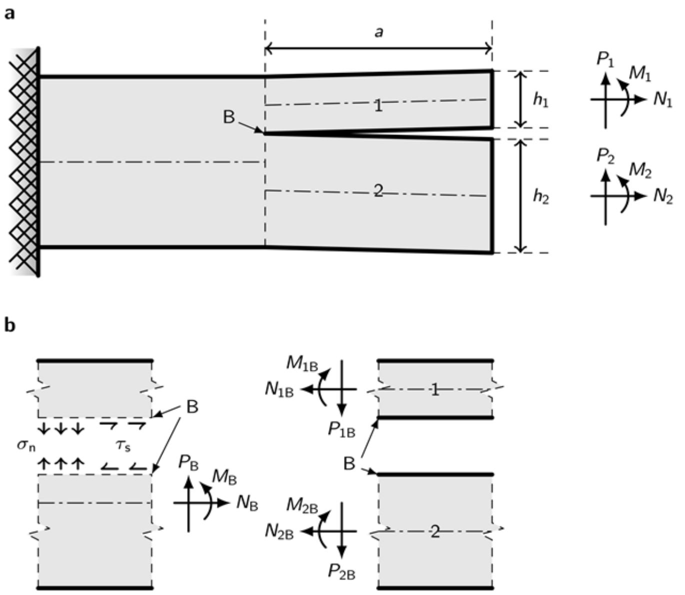

Supplementary Figure 1 | Double cantilever beam. a, Geometry and loading conditions. **b,** Details local to the crack tip.

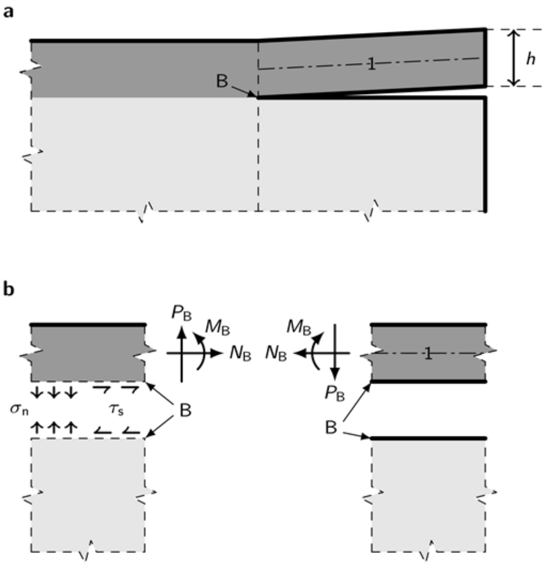

Supplementary Figure 2 | Blister test interface crack. a, Thin layer on a thick substrate. **b,** Effective crack tip forces and bending moments.

Supplementary Tables

Supplementary Table 1 | Adhesion toughness of monolayer graphene films with λ based on $p\delta$.

	Koenig et al.'s measurements[19]			Present mechanical model			
	$p\,(\mathrm{MPa})$	$\delta\,(\mu\mathrm{m})$	$R_\mathrm{B}\,(\mu\mathrm{m})$	λ based on $p\delta$	$\eta = G_\mathrm{S}/G_\mathrm{J}$	$\rho = G_\mathrm{II}/G_\mathrm{I}$	$G_\mathrm{c}\,(\mathrm{J\ m^{-2}})$
$p_0 = 3.18\,\mathrm{MPa}$	1.709	0.363	2.492	0	0	2.319	0.404
	1.514	0.396	2.710	0	0	2.319	0.391
	1.267	0.463	2.934	0	0	2.319	0.382
	1.096	0.496	3.171	0	0	2.319	0.354
Group average				0	0	2.319	0.383
$p_0 = 3.55\,\mathrm{MPa}$	1.648	0.405	2.756	0	0	2.319	0.435
	1.429	0.456	2.947	0	0	2.319	0.425
	1.242	0.493	3.168	0	0	2.319	0.399
Group average				0	0	2.319	0.420
$p_0 = 3.95\,\mathrm{MPa}$	1.632	0.437	2.964	0	0	2.319	0.465
	1.547	0.466	3.021	0	0	2.319	0.470
	1.320	0.509	3.252	0	0	2.319	0.438
Group average				0	0	2.319	0.458
$p_0 = 4.10\,\mathrm{MPa}$	1.494	0.475	3.208	0	0	2.319	0.462
	1.429	0.502	3.376	0	0	2.319	0.468
	1.255	0.514	3.513	0	0	2.319	0.420
Group average				0	0	2.319	0.450
Overall average				0	0	2.319	0.424
Koenig et al.[19]						2.319	0.450

Supplementary Table 2 | Adhesion toughness of two-layer graphene films with λ based on $p\delta$.

	Koenig et al.'s measurements[19]			Present mechanical model			
	$p\,(\mathrm{MPa})$	$\delta\,(\mu\mathrm{m})$	$R_\mathrm{B}\,(\mu\mathrm{m})$	λ based on $p\delta$	$\eta = G_\mathrm{S}/G_\mathrm{J}$	$\rho = G_\mathrm{II}/G_\mathrm{I}$	$G_\mathrm{c}\,(\mathrm{J\ m^{-2}})$
$p_0 = 3.25\,\mathrm{MPa}$	1.684	0.288	2.401	0.213	0.200	1.393	0.379
	1.471	0.319	2.573	0.211	0.198	1.398	0.367
	1.284	0.345	2.738	0.208	0.195	1.407	0.345
Group average				0.211	0.198	1.400	0.364
$p_0 = 3.67\,\mathrm{MPa}$	1.380	0.341	2.830	0.212	0.199	1.398	0.368
	1.189	0.376	2.978	0.209	0.196	1.406	0.348
	1.085	0.407	3.146	0.208	0.195	1.408	0.344
Group average				0.210	0.197	1.404	0.353
$p_0 = 4.35\,\mathrm{MPa}$	1.076	0.456	3.322	0.214	0.201	1.391	0.384
	0.901	0.542	3.467	0.214	0.201	1.392	0.382
	0.756	0.583	3.679	0.208	0.195	1.408	0.343
Group average				0.212	0.199	1.397	0.370
Overall average				0.211	0.198	1.400	0.362
Koenig et al.[19]						2.319	

Supplementary Table 3 | Adhesion toughness of three-layer graphene films with λ based on $p\delta$.

	Koenig et al.'s measurements[19]			Present mechanical model			
	$p\,(\mathrm{MPa})$	$\delta\,(\mu\mathrm{m})$	$R_\mathrm{B}\,(\mu\mathrm{m})$	λ based on $p\delta$	$\eta = G_\mathrm{S}/G_\mathrm{J}$	$\rho = G_\mathrm{II}/G_\mathrm{I}$	$G_\mathrm{c}\,(\mathrm{J\ m^{-2}})$
$p_0 = 3.25\,\mathrm{MPa}$	1.623	0.280	2.467	0.259	0.250	1.267	0.370
	1.376	0.339	2.615	0.261	0.252	1.263	0.381
Group average				0.260	0.251	1.265	0.375
$p_0 = 3.67\,\mathrm{MPa}$	1.425	0.334	2.862	0.262	0.254	1.259	0.389
$p_0 = 4.35\,\mathrm{MPa}$	1.210	0.411	3.286	0.265	0.257	1.252	0.407
	1.020	0.478	3.405	0.264	0.255	1.255	0.399
Group average				0.265	0.256	1.254	0.403
Overall average				0.262	0.254	1.259	0.389
Koenig et al.[19]						2.319	

Supplementary Table 4 | Adhesion toughness of four-layer graphene films with λ based on $p\delta$.

	Koenig et al.'s measurements[19]			Present mechanical model			
	$p\,(\mathrm{MPa})$	$\delta\,(\mu\mathrm{m})$	$R_\mathrm{B}\,(\mu\mathrm{m})$	λ based on $p\delta$	$\eta = G_\mathrm{S}/G_\mathrm{J}$	$\rho = G_\mathrm{II}/G_\mathrm{I}$	$G_\mathrm{c}\,(\mathrm{J\ m^{-2}})$
$p_0 = 3.25\,\mathrm{MPa}$	1.535	0.265	2.664	0.258	0.249	1.270	0.331
	1.420	0.271	2.845	0.254	0.245	1.280	0.312
Group average				0.256	0.247	1.275	0.322
$p_0 = 3.67\,\mathrm{MPa}$	1.407	0.319	2.998	0.264	0.256	1.254	0.367
$p_0 = 4.35\,\mathrm{MPa}$	1.118	0.414	3.513	0.266	0.258	1.249	0.380
Overall average				0.261	0.252	1.263	0.348
Koenig et al.[19]						2.319	

Supplementary Table 5 | Adhesion toughness of five-layer graphene films with λ based on $p\delta$.

	Koenig et al.'s measurements[19]			Present mechanical model			
	$p\,(\mathrm{MPa})$	$\delta\,(\mu\mathrm{m})$	$R_\mathrm{B}\,(\mu\mathrm{m})$	λ based on $p\delta$	$\eta = G_\mathrm{S}/G_\mathrm{J}$	$\rho = G_\mathrm{II}/G_\mathrm{I}$	$G_\mathrm{c}\,(\mathrm{J\ m^{-2}})$
$p_0 = 3.25\,\mathrm{MPa}$	1.700	0.244	2.459	0.253	0.244	1.283	0.336
	1.621	0.252	2.587	0.252	0.243	1.285	0.331
	1.417	0.305	2.686	0.256	0.247	1.276	0.351
Group average				0.254	0.244	1.281	0.339
$p_0 = 3.67\,\mathrm{MPa}$	1.596	0.276	2.861	0.257	0.248	1.273	0.358
	1.517	0.289	2.961	0.257	0.248	1.273	0.356
	1.430	0.306	3.017	0.257	0.247	1.274	0.356
Group average				0.257	0.248	1.273	0.357
$p_0 = 4.35\,\mathrm{MPa}$	1.297	0.376	3.276	0.264	0.255	1.256	0.399
	1.181	0.384	3.372	0.259	0.250	1.268	0.369
	1.056	0.436	3.483	0.260	0.251	1.265	0.375
Group average				0.261	0.252	1.263	0.381
Overall average				0.257	0.248	1.272	0.359
Koenig et al.[19]						2.319	

Supplementary Table 6 | Alternative calculations for the adhesion toughness of monolayer graphene films with λ based on pR_B.

	Koenig et al.'s measurements[19]			Present mechanical model			
	p (MPa)	δ (μm)	R_B (μm)	λ based on pR_B	$\eta = G_S/G_J$	$\rho = G_{II}/G_I$	G_c (J m^{-2})
$p_0 = 3.18$ MPa	1.709	0.363	2.492	0	0	2.319	0.404
	1.514	0.396	2.710	0	0	2.319	0.391
	1.267	0.463	2.934	0	0	2.319	0.382
	1.096	0.496	3.171	0	0	2.319	0.354
Group average				0	0	2.319	0.383
$p_0 = 3.55$ MPa	1.648	0.405	2.756	0	0	2.319	0.435
	1.429	0.456	2.947	0	0	2.319	0.425
	1.242	0.493	3.168	0	0	2.319	0.399
Group average				0	0	2.319	0.420
$p_0 = 3.95$ MPa	1.632	0.437	2.964	0	0	2.319	0.465
	1.547	0.466	3.021	0	0	2.319	0.470
	1.320	0.509	3.252	0	0	2.319	0.438
Group average				0	0	2.319	0.458
$p_0 = 4.10$ MPa	1.494	0.475	3.208	0	0	2.319	0.462
	1.429	0.502	3.376	0	0	2.319	0.468
	1.255	0.514	3.513	0	0	2.319	0.420
Group average				0	0	2.319	0.450
Overall average				0	0	2.319	0.424
Koenig et al.[19]						2.319	0.450

Supplementary Table 7 | Alternative calculations for the adhesion toughness of two-layer graphene films with λ based on pR_B.

	Koenig et al.'s measurements[19]			Present mechanical model			
	p (MPa)	δ (μm)	R_B (μm)	λ based on pR_B	$\eta = G_S/G_J$	$\rho = G_{II}/G_I$	G_c (J m^{-2})
$p_0 = 3.25$ MPa	1.684	0.288	2.401	0.215	0.202	1.388	0.380
	1.471	0.319	2.573	0.210	0.197	1.401	0.366
	1.284	0.345	2.738	0.205	0.192	1.417	0.344
Group average				0.210	0.197	1.402	0.363
$p_0 = 3.67$ MPa	1.380	0.341	2.830	0.213	0.200	1.395	0.368
	1.189	0.376	2.978	0.206	0.193	1.415	0.347
	1.085	0.407	3.146	0.203	0.190	1.423	0.342
Group average				0.207	0.194	1.411	0.353
$p_0 = 4.35$ MPa	1.076	0.456	3.322	0.206	0.193	1.413	0.382
	0.901	0.542	3.467	0.197	0.184	1.441	0.377
	0.756	0.583	3.679	0.190	0.176	1.464	0.338
Group average				0.198	0.184	1.439	0.365
Overall average				0.205	0.192	1.417	0.360
Koenig et al.[19]						2.319	

Supplementary Table 8 | Alternative calculations for the adhesion toughness of three-layer graphene films with λ based on pR_B.

	Koenig et al.'s measurements[19]			Present mechanical model			
	p (MPa)	δ (μm)	R_B (μm)	λ based on pR_B	$\eta = G_S/G_J$	$\rho = G_{II}/G_I$	G_c (J m^{-2})
$p_0 = 3.25$ MPa	1.623	0.280	2.467	0.256	0.247	1.275	0.369
	1.376	0.339	2.615	0.247	0.237	1.298	0.376
Group average				0.252	0.242	1.287	0.373
$p_0 = 3.67$ MPa	1.425	0.334	2.862	0.258	0.249	1.271	0.387
$p_0 = 4.35$ MPa	1.210	0.411	3.286	0.256	0.246	1.276	0.404
	1.020	0.478	3.405	0.244	0.234	1.306	0.392
Group average				0.250	0.240	1.291	0.398
Overall average				0.252	0.243	1.285	0.386
Koenig et al.[19]						2.319	

Supplementary Table 9 | Alternative calculations for the adhesion toughness of four-layer graphene films with λ based on pR_B.

	Koenig et al.'s measurements[19]			Present mechanical model			
	p (MPa)	δ (μm)	R_B (μm)	λ based on pR_B	$\eta = G_S/G_J$	$\rho = G_{II}/G_I$	G_c (J m^{-2})
$p_0 = 3.25$ MPa	1.535	0.265	2.664	0.258	0.248	1.271	0.331
	1.420	0.271	2.845	0.257	0.247	1.274	0.313
Group average				0.257	0.248	1.273	0.322
$p_0 = 3.67$ MPa	1.407	0.319	2.998	0.260	0.251	1.264	0.366
$p_0 = 4.35$ MPa	1.118	0.414	3.513	0.254	0.245	1.280	0.375
Overall average				0.257	0.248	1.272	0.346
Koenig et al.[19]						2.319	

Supplementary Table 10 | Alternative calculations for the adhesion toughness of five-layer graphene films with λ based on pR_B.

	Koenig et al.'s measurements[19]			Present mechanical model			
	p (MPa)	δ (μm)	R_B (μm)	λ based on pR_B	$\eta = G_S/G_J$	$\rho = G_{II}/G_I$	G_c (J m^{-2})
$p_0 = 3.25$ MPa	1.700	0.244	2.459	0.249	0.239	1.294	0.335
	1.621	0.252	2.587	0.249	0.239	1.293	0.330
	1.417	0.305	2.686	0.241	0.230	1.314	0.347
Group average				0.246	0.236	1.300	0.337
$p_0 = 3.67$ MPa	1.596	0.276	2.861	0.256	0.247	1.275	0.358
	1.517	0.289	2.961	0.255	0.245	1.278	0.356
	1.430	0.306	3.017	0.252	0.242	1.287	0.354
Group average				0.254	0.245	1.280	0.356
$p_0 = 4.35$ MPa	1.297	0.376	3.276	0.250	0.240	1.290	0.394
	1.181	0.384	3.372	0.245	0.234	1.304	0.365
	1.056	0.436	3.483	0.239	0.227	1.322	0.368
Group average				0.245	0.234	1.305	0.376
Overall average				0.248	0.238	1.295	0.356
Koenig et al.[19]						2.319	

Supplementary Table 11 | Alternative calculations for the adhesion toughness of monolayer graphene films with λ based on δ/R_B.

	Koenig et al.'s measurements[19]			Present mechanical model			
	p (MPa)	δ (μm)	R_B (μm)	λ based on δ/R_B	$\eta = G_S/G_J$	$\rho = G_{II}/G_I$	G_c (J m^{-2})
$p_0 = 3.18$ MPa	1.709	0.363	2.492	0	0	2.319	0.404
	1.514	0.396	2.710	0	0	2.319	0.391
	1.267	0.463	2.934	0	0	2.319	0.382
	1.096	0.496	3.171	0	0	2.319	0.354
Group average				0	0	2.319	0.383
$p_0 = 3.55$ MPa	1.648	0.405	2.756	0	0	2.319	0.435
	1.429	0.456	2.947	0	0	2.319	0.425
	1.242	0.493	3.168	0	0	2.319	0.399
Group average				0	0	2.319	0.420
$p_0 = 3.95$ MPa	1.632	0.437	2.964	0	0	2.319	0.465
	1.547	0.466	3.021	0	0	2.319	0.470
	1.320	0.509	3.252	0	0	2.319	0.438
Group average				0	0	2.319	0.458
$p_0 = 4.10$ MPa	1.494	0.475	3.208	0	0	2.319	0.462
	1.429	0.502	3.376	0	0	2.319	0.468
	1.255	0.514	3.513	0	0	2.319	0.420
Group average				0	0	2.319	0.450
Overall average				0	0	2.319	0.424
Koenig et al.[19]						2.319	0.450

Supplementary Table 12 | Alternative calculations for the adhesion toughness of two-layer graphene films with λ based on δ/R_B.

	Koenig et al.'s measurements[19]			Present mechanical model			
	$p\,(\mathrm{MPa})$	$\delta\,(\mu\mathrm{m})$	$R_B\,(\mu\mathrm{m})$	λ based on δ/R_B	$\eta = G_S/G_J$	$\rho = G_{II}/G_I$	$G_c\,(\mathrm{J\,m^{-2}})$
$p_0 = 3.25\,\mathrm{MPa}$	1.684	0.288	2.401	0.208	0.194	1.410	0.378
	1.471	0.319	2.573	0.215	0.202	1.389	0.368
	1.284	0.345	2.738	0.218	0.206	1.379	0.348
Group average				0.213	0.201	1.393	0.364
$p_0 = 3.67\,\mathrm{MPa}$	1.380	0.341	2.830	0.209	0.195	1.407	0.367
	1.189	0.376	2.978	0.219	0.206	1.378	0.351
	1.085	0.407	3.146	0.224	0.212	1.362	0.349
Group average				0.217	0.204	1.382	0.356
$p_0 = 4.35\,\mathrm{MPa}$	1.076	0.456	3.322	0.238	0.226	1.324	0.392
	0.901	0.542	3.467	0.271	0.263	1.239	0.402
	0.756	0.583	3.679	0.274	0.267	1.230	0.364
Group average				0.261	0.252	1.264	0.386
Overall average				0.230	0.219	1.346	0.369
Koenig et al.[19]						2.319	

Supplementary Table 13 | Alternative calculations for the adhesion toughness of three-layer graphene films with λ based on δ/R_B.

	Koenig et al.'s measurements[19]			Present mechanical model			
	$p\,(\mathrm{MPa})$	$\delta\,(\mu\mathrm{m})$	$R_B\,(\mu\mathrm{m})$	λ based on δ/R_B	$\eta = G_S/G_J$	$\rho = G_{II}/G_I$	$G_c\,(\mathrm{J\,m^{-2}})$
$p_0 = 3.25\,\mathrm{MPa}$	1.623	0.280	2.467	0.269	0.261	1.243	0.373
	1.376	0.339	2.615	0.307	0.304	1.154	0.397
Group average				0.288	0.283	1.199	0.385
$p_0 = 3.67\,\mathrm{MPa}$	1.425	0.334	2.862	0.276	0.269	1.225	0.394
$p_0 = 4.35\,\mathrm{MPa}$	1.210	0.411	3.286	0.296	0.292	1.178	0.419
	1.020	0.478	3.405	0.332	0.334	1.099	0.424
Group average				0.314	0.313	1.139	0.421
Overall average				0.296	0.292	1.180	0.401
Koenig et al.[19]						2.319	

Supplementary Table 14 | Alternative calculations for the adhesion toughness of four-layer graphene films with λ based on δ/R_B .

	Koenig et al.'s measurements[19]			Present mechanical model			
	p (MPa)	δ (μm)	R_B (μm)	λ based on δ/R_B	$\eta = G_S/G_J$	$\rho = G_{II}/G_I$	G_c (J m^{-2})
$p_0 = 3.25$ MPa	1.535	0.265	2.664	0.259	0.250	1.268	0.331
	1.420	0.271	2.845	0.248	0.238	1.297	0.310
Group average				0.253	0.244	1.282	0.321
$p_0 = 3.67$ MPa	1.407	0.319	2.998	0.277	0.270	1.223	0.371
$p_0 = 4.35$ MPa	1.118	0.414	3.513	0.307	0.304	1.154	0.393
Overall average				0.273	0.265	1.236	0.352
Koenig et al.[19]						2.319	

Supplementary Table 15 | Alternative calculations for the adhesion toughness of five-layer graphene films with λ based on δ/R_B .

	Koenig et al.'s measurements[19]			Present mechanical model			
	p (MPa)	δ (μm)	R_B (μm)	λ based on δ/R_B	$\eta = G_S/G_J$	$\rho = G_{II}/G_I$	G_c (J m^{-2})
$p_0 = 3.25$ MPa	1.700	0.244	2.459	0.267	0.259	1.248	0.340
	1.621	0.252	2.587	0.262	0.253	1.261	0.334
	1.417	0.305	2.686	0.305	0.302	1.158	0.367
Group average				0.278	0.271	1.222	0.347
$p_0 = 3.67$ MPa	1.596	0.276	2.861	0.259	0.250	1.267	0.359
	1.517	0.289	2.961	0.262	0.254	1.259	0.358
	1.430	0.306	3.017	0.273	0.265	1.234	0.361
Group average				0.265	0.256	1.253	0.359
$p_0 = 4.35$ MPa	1.297	0.376	3.276	0.309	0.306	1.150	0.415
	1.181	0.384	3.372	0.306	0.304	1.156	0.385
	1.056	0.436	3.483	0.337	0.339	1.091	0.402
Group average				0.317	0.316	1.132	0.401
Overall average				0.287	0.281	1.203	0.369
Koenig et al.[19]						2.319	

Supplementary Notes

Supplementary Note 1. Mixed-mode partition theory

During the last decade or so, the authors and their colleagues have developed an orthogonal pure mode partition methodology for partitioning mixed-mode 1D interface fractures in layered composite materials into their pure mode components. One example of 1D interface fracture is the circular blister fracture of thin films, which consists of only the mode I and II fracture modes. 1D interface fracture can be readily represented by a double cantilever beam (DCB) of unit width[1,2], as shown in Supplementary Fig. 1. Its loading conditions consist of tip bending moments per unit width, M_1 and M_2, tip axial forces per unit width, N_1 and N_2, and tip through-thickness shear forces per unit width, P_1 and P_2. Supplementary Fig. 1b shows the internal loads at the crack tip and the sign convention of the interface normal stress σ_{n} and shear stress τ_{s}. Extensive analytical and numerical studies have been carried out to prove the validity of the methodology[3–12] and various independent experimental test results have been used to assess the methodology[12–17]. It is found to be sound and the development is clear and thorough. A detailed explanation of the methodology[3–12]—even just the aspects that are closely-related to the present work—is not possible here. Therefore, in order to focus on the present work, only the most essential parts are given for physical understanding in what follows.

Based on the well-known virtual crack closure technique and linear elastic fracture mechanics, the mode I and mode II energy release rates (ERRs) can be written as

$$G_{\mathrm{I}} = \lim_{\Delta \to 0} \frac{F_{\mathrm{op}} d_{\mathrm{op}}}{2\Delta} \tag{1}$$

$$G_{\mathrm{II}} = \lim_{\Delta \to 0} \frac{d_{\mathrm{sh}} F_{\mathrm{sh}}}{2\Delta} \tag{2}$$

in which F_{op} and d_{op} represent the crack tip opening force per unit width and opening displacement respectively; F_{sh} and d_{sh} represent the crack tip interface shearing force per unit width and displacement respectively; and Δ represents the crack extension length. Supplementary Eqs. (1) and (2) can be written in the following forms[7–10] based on 2D elasticity:

$$G_{\mathrm{I}} = c_{\mathrm{I}}\left(M_{1\mathrm{B}} - \frac{M_{2\mathrm{B}}}{\beta_{1\text{-}2\mathrm{D}}} - \frac{N_{1\mathrm{B}}}{\beta_{2\text{-}2\mathrm{D}}} - \frac{N_{2\mathrm{B}}}{\beta_{3\text{-}2\mathrm{D}}} - \frac{P_{1\mathrm{B}}}{\beta_{4\text{-}2\mathrm{D}}} - \frac{P_{2\mathrm{B}}}{\beta_{5\text{-}2\mathrm{D}}}\right) \times \left(M_{1\mathrm{B}} - \frac{M_{2\mathrm{B}}}{\beta'_{1\text{-}2\mathrm{D}}} - \frac{N_{1\mathrm{B}}}{\beta'_{2\text{-}2\mathrm{D}}} - \frac{N_{2\mathrm{B}}}{\beta'_{3\text{-}2\mathrm{D}}} - \frac{P_{1\mathrm{B}}}{\beta'_{4\text{-}2\mathrm{D}}} - \frac{P_{2\mathrm{B}}}{\beta'_{5\text{-}2\mathrm{D}}}\right) \quad (3)$$

$$G_{\mathrm{II}} = c_{\mathrm{II}}\left(M_{1\mathrm{B}} - \frac{M_{2\mathrm{B}}}{\theta_{1\text{-}2\mathrm{D}}} - \frac{N_{1\mathrm{B}}}{\theta_{2\text{-}2\mathrm{D}}} - \frac{N_{2\mathrm{B}}}{\theta_{3\text{-}2\mathrm{D}}} - \frac{P_{1\mathrm{B}}}{\theta_{4\text{-}2\mathrm{D}}} - \frac{P_{2\mathrm{B}}}{\theta_{5\text{-}2\mathrm{D}}}\right) \times \left(M_{1\mathrm{B}} - \frac{M_{2\mathrm{B}}}{\theta'_{1\text{-}2\mathrm{D}}} - \frac{N_{1\mathrm{B}}}{\theta'_{2\text{-}2\mathrm{D}}} - \frac{N_{2\mathrm{B}}}{\theta'_{3\text{-}2\mathrm{D}}} - \frac{P_{1\mathrm{B}}}{\theta'_{4\text{-}2\mathrm{D}}} - \frac{P_{2\mathrm{B}}}{\theta'_{5\text{-}2\mathrm{D}}}\right) \quad (4)$$

By comparing Supplementary Eq. (3) with Supplementary Eq. (1), it is seen that the terms in the first and second brackets of Supplementary Eq. (3) correspond to F_{op} and d_{op} respectively, which are linearly proportional to the crack tip loads. This is required by linear elastic fracture mechanics. The crack tip loads consist of the bending moments per unit width, $M_{1\mathrm{B}}$ and $M_{2\mathrm{B}}$, the axial forces per unit width, $N_{1\mathrm{B}}$ and $N_{2\mathrm{B}}$, and the through-thickness shear forces per unit width, $P_{1\mathrm{B}}$ and $P_{2\mathrm{B}}$. The coefficients c_{I} and c_{II} are constants. The parameters $\beta_{i\text{-}2\mathrm{D}}$ and $\beta'_{i\text{-}2\mathrm{D}}$ (with $i = 1,2,3,4,5$) are independent of the crack tip loads and dependent on the DCB material properties, interface properties, fracture location, crack extension size, etc. They are called pure mode II modes for reasons best shown by example: When the crack tip loading conditions are $M_{1\mathrm{B}} = 1$, $M_{2\mathrm{B}} = \beta_{1\text{-}2\mathrm{D}}$, $N_{1\mathrm{B}} = 0$, $N_{2\mathrm{B}} = 0$, $P_{1\mathrm{B}} = 0$, $P_{2\mathrm{B}} = 0$, or in a vector form $\{M_{1\mathrm{B}} \quad M_{2\mathrm{B}} \quad N_{1\mathrm{B}} \quad N_{2\mathrm{B}} \quad P_{1\mathrm{B}} \quad P_{2\mathrm{B}}\}^{\mathrm{T}} = \{1 \quad \beta_{1-2\mathrm{D}} \quad 0 \quad 0 \quad 0 \quad 0\}^{\mathrm{T}}$ with the superscript T denoting transposition, the first bracket in Supplementary Eq. (3), corresponding to F_{op}, equals zero and therefore mode I ERR $G_{\mathrm{I}} = 0$; hence, $\beta_{1\text{-}2\mathrm{D}}$ is called a pure mode II mode due to zero crack tip opening force. Similarly, $\beta_{i\text{-}2\mathrm{D}}$ (with $i = 2,3,4,5$) are also called pure mode II modes due to zero crack tip opening force. Using the equivalent explanation, $\beta'_{i\text{-}2\mathrm{D}}$ (with $i = 1,2,3,4,5$) are called pure mode II modes due to zero crack tip opening displacement. It is worth noting that $\beta_{i\text{-}2\mathrm{D}}$ and $\beta'_{i\text{-}2\mathrm{D}}$ (with $i = 1,2,3,4,5$) are different from each other in the case of bi-material interfaces because the material mismatch causes a phase difference between the variations of stress and displacement[8,9,18], and they are also crack tip extension size-dependent[8,9,18].

Similarly, By comparing Supplementary Eq. (4) with Supplementary Eq. (2), it is seen that the terms in the first and second brackets of Supplementary Eq. (4) correspond to d_{sh} and F_{sh} respectively. By using the same explanation as above, $\theta_{i\text{-}2\mathrm{D}}$ (with $i = 1,2,3,4,5$) are called pure

mode I modes due to zero crack tip shearing displacement, and $\theta'_{i\text{-2D}}$ (with $i = 1,2,3,4,5$) are called pure mode I modes due to zero crack tip shearing force. Again, $\theta_{i\text{-2D}}$ and $\theta'_{i\text{-2D}}$ (with $i = 1,2,3,4,5$) are different from each other in the case of bi-material interfaces because the material mismatch causes a phase difference between variations of stress and displacement[8,9,18] and they are also crack tip extension size dependent[8,9,18]. In the case of homogeneous interfaces, $\beta_{i\text{-2D}}$ and $\beta'_{i\text{-2D}}$ (with $i = 1,2,3,4,5$) are equal to each other, and $\theta_{i\text{-2D}}$ and $\theta'_{i\text{-2D}}$ (with $i = 1,2,3,4,5$) are also equal to each other because then there is no phase difference between variations of stress and displacement[8,9,18] and they are also independent of crack tip extension size[8,9,18]. Then, Supplementary Eqs. (3) and (4) become

$$G_{\text{I}} = c_{\text{I}}\left(M_{1\text{B}} - \frac{M_{2\text{B}}}{\beta_{1\text{-2D}}} - \frac{N_{1\text{B}}}{\beta_{2\text{-2D}}} - \frac{N_{2\text{B}}}{\beta_{3\text{-2D}}} - \frac{P_{1\text{B}}}{\beta_{4\text{-2D}}} - \frac{P_{2\text{B}}}{\beta_{5\text{-2D}}}\right)^2 \quad (5)$$

$$G_{\text{II}} = c_{\text{II}}\left(M_{1\text{B}} - \frac{M_{2\text{B}}}{\theta_{1\text{-2D}}} - \frac{N_{1\text{B}}}{\theta_{2\text{-2D}}} - \frac{N_{2\text{B}}}{\theta_{3\text{-2D}}} - \frac{P_{1\text{B}}}{\theta_{4\text{-2D}}} - \frac{P_{2\text{B}}}{\theta_{5\text{-2D}}}\right)^2 \quad (6)$$

Furthermore, in the case of isotropic materials, Supplementary Eqs. (5) and (6) reduce to[7,10]

$$G_{\text{I}} = c_{\text{I}}\left(M_{1\text{B}} - \frac{M_{2\text{B}}}{\beta_{1\text{-2D}}} - \frac{N_{1\text{Be}}}{\beta_{2\text{-2D}}} - \frac{P_{1\text{B}}}{\beta_{3\text{-2D}}} - \frac{P_{2\text{B}}}{\beta_{4\text{-2D}}}\right)^2 \quad (7)$$

$$G_{\text{II}} = c_{\text{II}}\left(M_{1\text{B}} - \frac{M_{2\text{B}}}{\theta_{1\text{-2D}}} - \frac{N_{1\text{Be}}}{\theta_{2\text{-2D}}} - \frac{P_{1\text{B}}}{\theta_{3\text{-2D}}} - \frac{P_{2\text{B}}}{\theta_{4\text{-2D}}}\right)^2 \quad (8)$$

where $N_{1\text{Be}} = N_{1\text{B}} - N_{2\text{B}}/\gamma$. The pure modes, $\theta_{i\text{-2D}}$ and $\beta_{i\text{-2D}}$ (with $i = 1,2,3,4$), in Supplementary Eqs. (7) and (8) were derived by the authors[7,10] using a powerful orthogonal pure mode methodology and have been thoroughly verified against numerical simulations (interested readers are advised to read refs. 7 and 10). They are recorded below.

$$\theta_{1\text{-2D}} = -\gamma^2 - \frac{\bar{c}_\theta^{\,2}\gamma^2\left(1-\gamma^2\right)}{\bar{c}_\theta^{\,2}\gamma^2 + \alpha(1+\gamma)^2} \quad (9)$$

$$\beta_{1\text{-2D}} = \frac{\gamma^2(3+\gamma)}{1+3\gamma} - \frac{3\bar{c}_\theta^{\,2}\gamma^2\left(1-\gamma^2\right)}{(1+3\gamma)\left[\bar{c}_\theta^{\,2} + \alpha(1+3\gamma)\right]} \quad (10)$$

$$\theta_{2\text{-2D}} = -\frac{6}{h_1} - \frac{6\bar{c}_\theta^{\,2}\gamma(1-\gamma)}{h_1\left[\bar{c}_\theta^{\,2}\left(1-\gamma+\gamma^2\right) + \alpha(1+\gamma)^2\right]} \quad (11)$$

$$\beta_{2\text{-2D}} = \begin{cases} \dfrac{2(3+\gamma)}{h_1(\gamma-1)} + \dfrac{2\bar{c}_\theta^{\,2}\gamma}{h_1\alpha(\gamma-1)} & \text{if } \gamma \neq 1 \\ \infty & \text{if } \gamma = 1 \end{cases} \tag{12}$$

$$\theta_{3\text{-2D}} = \frac{\theta_{1\text{-2D}}\left(1+\beta_{\text{P-2D}}\right)}{\left(\beta_{1\text{-2D}} - \theta_{1\text{-2D}}\right)\left(G_{\beta_{\text{P-2D}}}/G_{\beta_{1\text{-2D}}}\right)^{1/2}} \tag{13}$$

$$\beta_{3\text{-2D}} = \frac{\beta_{1\text{-2D}}\left(1+\beta_{\text{P-2D}}\right)}{\beta_{\text{P-2D}}\left(\theta_{1\text{-2D}} - \beta_{1\text{-2D}}\right)\left(G_{\theta_{\text{P-2D}}}/G_{\theta_{1\text{-2D}}}\right)^{1/2}} \tag{14}$$

$$\theta_{4\text{-2D}} = -\theta_{\text{P-2D}}\theta_{3\text{-2D}} \tag{15}$$

$$\beta_{4\text{-2D}} = -\beta_{\text{P-2D}}\beta_{3\text{-2D}} \tag{16}$$

The thickness ratio of the beams h_2/h_1 is denoted by γ; also $\bar{c}_\theta \approx 6/5$ and $\alpha = \bar{c}_\theta^{\,e}$ with $e = \left[(1+\gamma)^3/(1+\gamma^3)\right]^{1/2}$. For through-thickness shear forces at the crack tip, $P_{1\text{B}}$ and $P_{2\text{B}}$ (with $M_{1\text{B}} = M_{2\text{B}} = N_{1\text{B}} = N_{2\text{B}} = 0$), the pure modes $\theta_{\text{P-2D}}$ and $\beta_{\text{P-2D}}$ are

$$\left(\theta_{\text{P-2D}}, \beta_{\text{P-2D}}\right) = \left(-1, \gamma\exp\left(-1.986060\operatorname{atanh}\left(0.563483\gamma_i\right)\right)\right) \tag{17}$$

where $\gamma_{\text{i}} = \log_{10}(1/\gamma)$. The remaining parameters in Supplementary Eqs. (7), (8), (13) and (14) are

$$c_{\text{I}} = G_{\theta_{1\text{-2D}}}\left(1 - \frac{\theta_{1\text{-2D}}}{\beta_{1\text{-2D}}}\right)^{-2} \quad , \quad c_{\text{II}} = G_{\beta_{1\text{-2D}}}\left(1 - \frac{\beta_{1\text{-2D}}}{\theta_{1\text{-2D}}}\right)^{-2} \tag{18}$$

$$G_{\theta_{1\text{-2D}}} = \frac{6}{b^2 h_1^{\,3}\bar{E}}\left(1 + \frac{\theta_{1\text{-2D}}^{\,2}}{\gamma^3} - \frac{\left(1+\theta_{1\text{-2D}}\right)^2}{(1+\gamma)^3}\right) \tag{19}$$

$$G_{\beta_{1\text{-2D}}} = \frac{6}{b^2 h_1^{\,3}\bar{E}}\left(1 + \frac{\beta_{1\text{-2D}}^{\,2}}{\gamma^3} - \frac{\left(1+\beta_{1\text{-2D}}\right)^2}{(1+\gamma)^3}\right) \tag{20}$$

$$G_{\theta_{\text{P-2D}}} = \frac{1}{2b^2 h_1\bar{E}\kappa(\gamma)}\left(1 + \frac{\theta_{\text{P-2D}}^{\,2}}{\gamma}\right) \tag{21}$$

$$G_{\beta_{\text{P-2D}}} = \frac{1}{2b^2 h_1\bar{E}\kappa(\gamma)}\left(1 + \frac{\beta_{\text{P-2D}}^{\,2}}{\gamma} - \frac{\left(1+\beta_{\text{P-2D}}\right)^2}{1+\gamma}c(\gamma)\right) \tag{22}$$

where $b = 1$ is the unit width of the beam, and $\bar{E} = E$ for plane stress or $\bar{E} = E/(1-\nu^2)$ for plane strain, with E being the Young's modulus of the beam and ν being the Poisson's ratio. The two thickness ratio γ-dependent correction factors, namely the through-thickness shear

correction factor $\kappa(\gamma)$ and the pure-mode-II ERR correction factor $c(\gamma)$, can be calculated using the following elegant expressions:

$$\kappa(\gamma) = 0.135535 + 0.047743\exp\left(-1.390931\gamma_i^{\,2}\right) \tag{23}$$

$$c(\gamma) = \frac{\left(1+\beta_{\text{P-2D}}^{\,2}/\gamma\right)\left(1+\gamma\right)}{\left(1+\beta_{\text{P-2D}}\right)^2} C_{\text{F}} \tag{24}$$

$$C_{\text{F}} = 1 - 0.070920\exp\left(-3.280135\gamma_i^{\,2}\right) \tag{25}$$

It is worth noting that in the absence of through-thickness shear forces, $P_{1\text{B}}$ and $P_{2\text{B}}$, Supplementary Eqs. (7) and (8) are extremely close to Suo & Hutchinson's 2D partitions[1,2]; that is, the pure modes in Supplementary Eqs. (9)–(12) are nearly identical to Suo & Hutchinson's pure modes[1,2] and are just presented in different forms.

Supplementary Note 2. Development of mixed-mode partition theory for thin film blister test

In the following, Supplementary Eqs. (7) and (8) are extended to the case of thin films in the blister test to determine the adhesion toughness, for example, the adhesion toughness of multilayer graphene films[19]. The substrate is treated as infinitely thick and the films as very thin, as shown in Supplementary Fig. 2a; therefore, the thickness ratio tends to infinity $\gamma \to \infty$. The authors' latest work on the mechanical behaviour of thin film spallation[14–17] shows that excellent agreement is achieved with experimental results[20–24] when the material mismatch between a film and its substrate is neglected. Furthermore, in these studies[14,15] slightly worse agreement was found with experimental results[20,21] when the mismatch[8,9] was taken into account. Therefore, the present work also neglects the material mismatch, and Supplementary Eqs. (7) and (8) become

$$G_{\text{I}} = c_{\text{I}}\left(M_{\text{B}} - \frac{N_{\text{B}}}{\beta_{2-2\text{D}}} - \frac{P_{\text{B}}}{\beta_{3-2\text{D}}}\right)^2 \tag{26}$$

$$G_{\text{II}} = c_{\text{II}}\left(M_{\text{B}} - \frac{N_{\text{B}}}{\theta_{2-2\text{D}}} - \frac{P_{\text{B}}}{\theta_{3-2\text{D}}}\right)^2 \tag{27}$$

where M_{B}, N_{B} and P_{B}, which are shown in Supplementary Fig. 2b, are the effective crack tip bending moment, axial force and shear force respectively. The pure modes $\theta_{2-2\text{D}}$ and $\beta_{2-2\text{D}}$ when $\gamma \to \infty$, based on Suo & Hutchinson[1,2], are

$$\left(\theta_{2-2\mathrm{D}}, \beta_{2-2\mathrm{D}}\right) = \left(-\frac{2.697}{h_1}, \frac{4.450}{h_1} \right) \tag{28}$$

where h_1 is the film thickness. Note that the authors' pure modes $\theta_{2-2\mathrm{D}}$ and $\beta_{2-2\mathrm{D}}$ in Supplementary Eqs. (11) and (12) also give very close values to Supplementary Eq. (28); however, in the present work, Supplementary Eq. (28) is used.

The coefficient parameters, c_{I} in Supplementary Eq. (26) and c_{II} in Supplementary Eq. (27), must be determined: In the absence of through-thickness shear force P_{B}, the total ERR is given by

$$G = \frac{6}{\bar{E}b^2h_1^3}\left(M_{\mathrm{B}}^2 + \frac{h_1^2}{12} N_{\mathrm{B}}^2 \right) \tag{29}$$

In the case of pure mode $\theta_{2-2\mathrm{D}}$, that is, with $N_{\mathrm{B}} = \theta_{2-2\mathrm{D}} M_{\mathrm{B}}$ and $P_{\mathrm{B}} = 0$, Supplementary Eqs. (26), (28) and (29) give

$$c_{\mathrm{I}} = 0.6227 \frac{6}{\bar{E}b^2h_1^3} \tag{30}$$

Similarly, in the case of pure mode $\beta_{2-2\mathrm{D}}$, that is, with $N_{\mathrm{B}} = \beta_{2-2\mathrm{D}} M_{\mathrm{B}}$ and $P_{\mathrm{B}} = 0$, Supplementary Eqs. (27), (28) and (29) give

$$c_{\mathrm{II}} = 0.3773 \frac{6}{\bar{E}b^2h_1^3} \tag{31}$$

The pure modes, $\beta_{3-2\mathrm{D}}$ in Supplementary Eq. (26) and $\theta_{3-2\mathrm{D}}$ in Supplementary Eq. (27), must also be determined: By rearranging Supplementary Eqs. (13) and (14), they become

$$\theta_{3\text{-}2\mathrm{D}} = \frac{\left(1 + \beta_{\mathrm{P}\text{-}2\mathrm{D}}\right)}{\left(\beta_{1\text{-}2\mathrm{D}}/\theta_{1\text{-}2\mathrm{D}} - 1\right)\left(G_{\beta_{\mathrm{P}\text{-}2\mathrm{D}}}/G_{\beta_{1\text{-}2\mathrm{D}}}\right)^{1/2}} \tag{32}$$

$$\beta_{3\text{-}2\mathrm{D}} = \frac{\left(1/\beta_{\mathrm{P}\text{-}2\mathrm{D}} + 1\right)}{\left(\theta_{1\text{-}2\mathrm{D}}/\beta_{1\text{-}2\mathrm{D}} - 1\right)\left(G_{\theta_{\mathrm{P}\text{-}2\mathrm{D}}}/G_{\theta_{1\text{-}2\mathrm{D}}}\right)^{1/2}} \tag{33}$$

Note that before Supplementary Eqs. (32) and (33) can be used in Supplementary Eqs. (26) and (27), which are for thin films, they must be reduced to the limit where $\gamma \to \infty$. To do this, each of the ratios $\theta_{1-2\mathrm{D}}/\beta_{1-2\mathrm{D}}$, $G_{\beta_{\mathrm{P}\text{-}2\mathrm{D}}}/G_{\beta_{1\text{-}2\mathrm{D}}}$ and $G_{\theta_{\mathrm{P}\text{-}2\mathrm{D}}}/G_{\theta_{1\text{-}2\mathrm{D}}}$ in Supplementary Eqs. (32) and (33) must also be reduced to this limit, and they are now each considered in turn.

The ratio $\theta_{1-2\mathrm{D}}/\beta_{1-2\mathrm{D}}$ in Supplementary Eqs. (32) and (33) when $\gamma \to \infty$ is determined first. In the general case with crack tip moments $M_{1\mathrm{B}}$ and $M_{2\mathrm{B}}$ only, the total ERR is given by

$$G = \frac{6}{\overline{E}b^2h_1^3}\left(M_{1B}^2 + \frac{M_{2B}^2}{\gamma^3} - \frac{(M_{1B}+M_{2B})^2}{(1+\gamma)^3}\right) \quad (34)$$

In the case of pure mode θ_{1-2D}, that is, with $M_{2B} = \theta_{1-2D}M_{1B}$ and $M_{1B} = 1$, Supplementary Eqs. (7), (30) and (34) give

$$1 + \frac{\theta_{1-2D}^2}{\gamma^3} - \frac{(1+\theta_{1-2D})^2}{(1+\gamma)^3} = 0.6227\left(1 - \frac{\theta_{1-2D}}{\beta_{1-2D}}\right)^2 \quad (35)$$

Similarly, in the case of pure mode β_{1-2D}, that is, with $M_{2B} = \beta_{1-2D}M_{1B}$ and $M_{1B} = 1$, Supplementary Eqs. (8), (31) and (34) give

$$1 + \frac{\beta_{1-2D}^2}{\gamma^3} - \frac{(1+\beta_{1-2D})^2}{(1+\gamma)^3} = 0.3773\left(1 - \frac{\beta_{1-2D}}{\theta_{1-2D}}\right)^2 \quad (36)$$

When $\gamma \to \infty$, Supplementary Eqs. (35) and (36) give

$$\frac{\theta_{1\text{-}2D}}{\beta_{1\text{-}2D}} = -0.6059 \quad (37)$$

Note that the pure modes in Supplementary Eq. (28) (refs. 1,2) are used in deriving Supplementary Eq. (37). When pure modes in Supplementary Eqs. (11) and (12), derived by the authors[7], are used, the ratio becomes $\theta_{1\text{-}2D}/\beta_{1\text{-}2D} = -75/121 \approx -0.6198$ which is very close to -0.6059 in Supplementary Eq. (37). In the present work, Supplementary Eq. (37) is used as it is believed to be more accurate.

The quantities $G_{\theta_{P\text{-}2D}}$, $G_{\beta_{P\text{-}2D}}$, $G_{\theta_{1\text{-}2D}}$ and $G_{\beta_{1\text{-}2D}}$ in Supplementary Eqs. (32) and (33) when $\gamma \to \infty$ are determined next. When $\gamma \to \infty$, Supplementary Eq. (23) gives $\kappa(\infty) = 0.135535$. Then, Supplementary Eqs. (17) and (21) give

$$G_{\theta_{P\text{-}2D}} = \frac{1}{2b^2h_1\overline{E}} \times \frac{1}{0.135535} \quad (38)$$

In the case of pure mode θ_{1-2D}, that is, with $M_{2B} = \theta_{1-2D}M_{1B}$ and $M_{1B} = 1$, Supplementary Eqs. (18), (30) and (37) give

$$G_{\theta_{1\text{-}2D}} = \frac{6}{b^2h_1^3\overline{E}} \times 1.6059 \quad (39)$$

In the case of pure mode β_{1-2D}, that is, with $M_{2B} = \beta_{1-2D}M_{1B}$ and $M_{1B} = 1$, Supplementary Eqs. (18), (31) and (37) give

$$G_{\beta_{1\text{-}2D}} = \frac{6}{b^2h_1^3\overline{E}} \times 2.6505 \quad (40)$$

When $\gamma \to \infty$, then $C_{\mathrm{F}}(\infty) = 1$ from Supplementary Eq. (25). Therefore, Supplementary Eq. (22) gives

$$G_{\beta_{\text{P-2D}}} = 0 \tag{41}$$

Now, substituting Supplementary Eqs. (17) and (37)–(41) into Supplementary Eqs. (32) and (33) gives $\theta_{\text{3-2D}}$ and $\beta_{\text{3-2D}}$ as

$$(\theta_{\text{3-2D}}, \beta_{\text{3-2D}}) = \left(\infty,\ -\frac{1.0063}{h_1} \right) \tag{42}$$

Note that as $\theta_{\text{3-2D}} = \infty$, the effective crack tip through-thickness shear force P_{B} only contributes to the mode I component of the ERR G_{I}.

All terms in Supplementary Eqs. (26) and (27) have now been derived. Again, it is important to note that the through-thickness shear force increases the mode I ERR component, and that this effect itself increases for thicker films, as shown by Supplementary Eqs. (26) and (42). Therefore, the thicker the film is, the lower the adhesion toughness. Supplementary Eqs. (26) and (27) have also been used[17] to study the adhesion toughness of thin photoresist films under linear bending with small deflection. The films were experimentally tested by Cao et al.[27] using the circular blister test. The experimental results indeed show the reduction of the adhesion toughness.

Now, Supplementary Eqs. (26) and (27) are applied to study the adhesion toughness of multilayer graphene films, such as those in Koenig et al.'s[19] work. It is noted that films can generally be under bending, stretching and shearing; however, when films are in the membrane limit (i.e. only under stretching), they are referred to as 'membranes'. In Koenig et al.'s[19] work, multilayer graphene films are in this membrane-stretching state, and the crack tip forces in Supplementary Eqs. (26) and (27) for a circular blister of radius R_{B} under a pressure load p are then given by[25,26]

$$\begin{aligned} M_{\mathrm{B}} &= \frac{nt}{4} \frac{\left(nEtp^2 R_{\mathrm{B}}^2\right)^{1/3}}{\left[3\left(1-\nu^2\right)\varphi(\nu)\right]^{1/2}} = \frac{nt}{4} \left(\frac{nEtp\delta}{3\left(1-\nu^2\right)\varphi(\nu) f(\nu)} \right)^{1/2} \\ &= \frac{nt}{4} \frac{nEt}{\left[3\left(1-\nu^2\right)\varphi(\nu)\right]^{1/2}} \left(\frac{\delta}{f(\nu) R_{\mathrm{B}}} \right)^2 \end{aligned} \tag{43}$$

$$N_{\mathrm{B}} = \left(nEtp^2 R_{\mathrm{B}}^2\right)^{1/3} \varphi(\nu) = \left(\frac{nEtp\delta}{f(\nu)} \right)^{1/2} \varphi(\nu) = nEt \left(\frac{\delta}{f(\nu) R_{\mathrm{B}}} \right)^2 \tag{44}$$

$$P_{\mathrm{B}} = \frac{1}{2} p R_{\mathrm{B}} \tag{45}$$

Note that a circular blister is now specifically studied; therefore $b=1$ as the forces in Supplementary Eqs. (43)–(45) are per unit width. The quantities n, E and t represent the number of graphene layers, the Young's modulus, and the thickness of monolayer graphene, respectively. The centre deflection δ, and the Poisson's ratio ν-dependent parameters, $f(\nu)$ and $\varphi(\nu)$, are given in Eqs. (1), (2) and (6) in the main article.

Note that various expressions for $f(\nu)$ and $\varphi(\nu)$ are reported in literature[28] due to different approximations being used in their derivations. Jensen's[25,26] total ERR results, however, are very close to Hencky's[29], as shown in the main article. Furthermore, Jensen's[25,26] total ERR results for monolayer membranes agree very well with Wang & Tong's[30] values from finite element simulations. Therefore, based on these considerations, Jensen's[25,26] expressions for $f(\nu)$ and $\varphi(\nu)$, along with Supplementary Eqs. (43)–(45) are used in the present work.

Substituting Supplementary Eqs. (43)–(45) into Supplementary Eqs. (26) and (27) gives

$$\begin{aligned} G_{\mathrm{I}} &= 0.6227\times\frac{6M_{\mathrm{B}}^{2}}{E(nt)^{3}}\left(1-\nu^{2}\right)\left(1-\frac{\chi(\nu)}{4.450}+\lambda\right)^{2} \\ &= 0.6227\times\frac{6M_{\mathrm{B}}^{2}}{E(nt)^{3}}\left(1-\nu^{2}\right)\left(0.7578-0.1429\nu+\lambda\right)^{2} \\ &= 0.6227\times\frac{1}{8}p\delta\frac{\left(0.7578-0.1429\nu+\lambda\right)^{2}}{\varphi(\nu)f(\nu)} \\ &= 0.6227\times\frac{1}{8}\left(\frac{p^{4}R_{\mathrm{B}}^{4}}{nEt}\right)^{1/3}\frac{\left(0.7578-0.1429\nu+\lambda\right)^{2}}{\varphi(\nu)} \\ &= 0.6227\times\frac{1}{8}\left(\frac{\delta}{R_{\mathrm{B}}}\right)^{4}\frac{nEt\left(0.7578-0.1429\nu+\lambda\right)^{2}}{\varphi(\nu)f^{4}(\nu)} \end{aligned} \tag{46}$$

$$\begin{aligned} G_{\mathrm{II}} &= 0.3773\times\frac{6M_{\mathrm{B}}^{2}}{E(nt)^{3}}\left(1-\nu^{2}\right)\left(1+\frac{\chi(\nu)}{2.697}\right)^{2} \\ &= 0.3773\times\frac{6M_{\mathrm{B}}^{2}}{E(nt)^{3}}\left(1-\nu^{2}\right)\left(1.400+0.2358\nu\right)^{2} \\ &= 0.3773\times\frac{1}{8}p\delta\frac{\left(1.400+0.2358\nu\right)^{2}}{\varphi(\nu)f(\nu)} \\ &= 0.3773\times\frac{1}{8}\left(\frac{p^{4}R_{\mathrm{B}}^{4}}{nEt}\right)^{1/3}\frac{\left(1.400+0.2358\nu\right)^{2}}{\varphi(\nu)} \\ &= 0.3773\times\frac{1}{8}\left(\frac{\delta}{R_{\mathrm{B}}}\right)^{4}\frac{nEt\left(1.400+0.2358\nu\right)^{2}}{\varphi(\nu)f^{4}(\nu)} \end{aligned} \tag{47}$$

where

$$\chi(\nu) = \frac{N_{\mathrm{B}} n t}{M_{\mathrm{B}}} = 4\left[3\left(1-\nu^2\right)\varphi^3(\nu)\right]^{1/2} \tag{48}$$

$$\lambda = \bar{\lambda} S(n) = -\frac{P_{\mathrm{B}}}{M_{\mathrm{B}}\beta_{3\text{-}2\mathrm{D}}} S(n) \tag{49}$$

with

$$\bar{\lambda} = -\frac{P_{\mathrm{B}}}{M_{\mathrm{B}}\beta_{3\text{-}2\mathrm{D}}} \tag{50}$$

and

$$S(n) = 1 - e^{1-n} \tag{51}$$

The origin of the parameter $\bar{\lambda}$ is obvious, but the origin of the factor $S(n)$ needs to be explained. Monolayer films are considered first. First, some pertinent observations: As mentioned earlier, Jensen's[25,26] total ERR results for monolayer membranes agree very well with Wang & Tong's[30] values from finite element simulations. Jensen, however, only included the contributions from the bending moment M_{B} and the axial force N_{B}, as given by Supplementary Eqs. (43) and (44) respectively. This indicates that the through-thickness shear force P_{B}, given by Supplementary Eq. (45), does not contribute to ERR for monolayer membranes. Furthermore, in other work by the authors[17], two scenarios are considered using the methodology developed in the present work: (1) linear bending of monolayer films at small deflection, including the through-thickness force P_{B}, and (2) membrane stretching of monolayer films at large deflection without including the through-thickness force P_{B}. The analytical predictions for the adhesion toughness between photoresist films and copper substrates are in excellent agreement with the experimental results[27]. The thicknesses of the photoresist films are 10 μm (for membrane stretching at large deflection), and 31 μm and 60 μm (both for linear bending at small deflection). This indicates that the through-thickness shear force has no effect on ERR for membrane stretching of monolayer films at large deflection, while it does have effect on ERR for linear bending of monolayer films at small deflection. Now, the explanation for these observations is given: In the case of linear bending at small deflection, through-thickness shear strain is produced by the through-thickness shear force. They together result in through-thickness shear strain energy and contribute to the ERR at crack tip. In the case of membrane stretching at large deflection, there is no through-thickness shear force in the membrane blister resulting in no through-thickness strain. Although transition from membrane stretching to combined bending, stretching and through-thickness shearing occurs near the crack tip, the through-thickness shear strain energy at crack

tip is still negligible. Therefore, the through-thickness shear force has no effect on ERR in the membrane limit for monolayer films.

Multilayer graphene membranes[19] are considered next. As before, for linear bending at small deflection, through-thickness shear force exists and produces through-thickness strain, resulting in extra ERR. In the membrane limit, it is expected that multilayer graphene films in the membrane region of a blister behave as a single layer because there is only membrane stretching. The transition from membrane stretching to combined bending, stretching and through-thickness shearing occurs near the crack tip. If a multilayer graphene film still behaves as a single layer in the transition region, as is the case for monolayer graphene membranes, the through-thickness shearing strain energy near the crack tip is still negligible, resulting in no ERR contribution; however, the transition can cause interlayer shearing and sliding in the transition region, at the crack tip in particular, the through-thickness shearing strain energy near the crack tip is no longer negligible, which does result in an ERR contribution. In fact, Koenig et al.'s[19] observations indeed demonstrate that for a typical interlayer shearing and sliding effect, the adhesion toughness has a large decrease between monolayer and two-layer graphene films, but then remains fairly constant afterwards.

The present work takes the interlayer shearing and sliding near crack tip into consideration by introducing the interlayer shearing and sliding factor $S(n)$. The arguments for the proposed expression for $S(n)$ in Supplementary Eq. (51) are as follows: (1) Obviously, no interlayer shearing and sliding can exist in monolayer graphene membranes, so $S(1)=0$. (2) The factor $S(n)$ must account for the fact mentioned above that adhesion toughness has a large decrease between monolayer and two-layer graphene films and remains fairly constant afterwards[19]. (3) From the view point of continuum mechanics, the converged value of $S(n)$ for multilayer graphene films is assumed to make a complete transition from membrane stretching to combined bending, stretching and shearing at the crack tip, so $S(\infty)=1$. The validity of $S(n)$ in Supplementary Eq. (51) is tested by experimental results in the main article.

In addition to the above explanation of $S(n)$, further interpretation of its mechanical meaning may be useful. The average through-thickness shear stress at crack tip is $\tau_{\mathrm{S}} = P_{\mathrm{B}}/(nt)$ and the effective average through-thickness shear strain at crack tip due to interlayer shearing and sliding is assumed to be γ_{S}, which can be estimated from the equation below.

$$c_{\mathrm{I}}\left(\frac{P_{\mathrm{B}}S(n)}{\beta_{3-2\mathrm{D}}}\right)^2=\frac{1}{2}P_{\mathrm{B}}\gamma_{\mathrm{S}} \tag{52}$$

The left-hand side is the ERR from Supplementary Eq. (26) with through-thickness shear force acting alone with the introduction of $S(n)$. The right-hand side is the effective through-thickness shear strain energy in a volume of dimensions $1\times1\times nt$. The effective through-thickness shear strain γ_{S} is then obtained as

$$\gamma_{\mathrm{S}}=2c_{\mathrm{I}}\left(\frac{S(n)}{\beta_{3-2\mathrm{D}}}\right)^2P_{\mathrm{B}} \tag{53}$$

and the effective through-thickness shear modulus G_{S} is then estimated as

$$G_{\mathrm{S}}=\frac{P_{\mathrm{B}}}{nt\gamma_{\mathrm{S}}} \tag{54}$$

Substituting Supplementary Eqs. (30), (42) and (53) into Supplementary Eq. (54) gives

$$G_{\mathrm{S}}=\frac{0.1355E}{(1-\nu^2)S^2(n)} \tag{55}$$

The value 0.1355 is $\kappa(\gamma)$ in Supplementary Eq. (23) when $\gamma\to\infty$. It is seen that the mechanical meaning of $S(n)$ is to introduce an effective through-thickness shear modulus G_{S} to account for the interlayer shearing and sliding. Note that G_{S} is just an effective value instead of the actual material property. This is similar to the case of classical plate theory in which the effective through-thickness shear modulus is infinitely large while the actual material property is finite.

The values of the λ parameter, based on Koenig et al.'s[19] measurements, are recorded in Supplementary Tables 1–15. There is a large decrease from monolayer to two-layer graphene films and then only a small decrease from two-layer to three-layer graphene films. For the three-, four- and five-layer graphene films, the values of the λ parameter are very close to each other. This shows the typical interlayer shearing and sliding behaviour.

Note that the interface between graphene films and their substrates is assumed to be a rigid interface[1–5,7–12], that is, it is assumed that no relative shearing and sliding displacement occurs before separation. This is consistent with Koenig et al.'s[19] work. The present methodology could, however, be extended to consider the shearing and sliding analytically by combining it with the authors' mixed-mode partition theory for non-rigid interface fractures[6]. Some complex mechanical behaviour such as wrinkling[31] can be caused by this type sliding, which will be considered in future work.

Finally, the critical interlayer shear stress for sliding is beyond the scope of the present work; however, the present methodology can be used to determine the mode I and II toughness between graphene layers using the blister test. The mode II toughness is considered to be the sliding toughness.

Supplementary References

1. Suo, Z. & Hutchinson, J. W. Interface crack between two elastic layers. *Int. J. Fract.* **43,** 1–18 (1990).
2. Hutchinson, J. W. & Suo, Z. Mixed mode cracking in layered materials. *Adv. Appl. Mech.* **92,** 63–191 (1991).
3. Wang, S. & Harvey, C. M. A theory of one-dimensional fracture. *Compos. Struct.* **94,** 758–767 (2012).
4. Wang, S. & Harvey, C. M. Mixed mode partition theories for one dimensional fracture. *Eng. Fract. Mech.* **79,** 329–352 (2012).
5. Harvey, C. M. & Wang, S. Mixed-mode partition theories for one-dimensional delamination in laminated composite beams. *Eng. Fract. Mech.* **96,** 737–759 (2012).
6. Wang, S., Harvey, C. M. & Guan, L. Partition of mixed modes in layered isotropic double cantilever beams with non-rigid cohesive interfaces. *Eng. Fract. Mech.* **111,** 1–25 (2013).
7. Harvey, C. M., Wood, J. D., Wang, S. & Watson, A. A novel method for the partition of mixed-mode fractures in 2D elastic laminated unidirectional composite beams, *Compos. Struct.* **116**, 589–594 (2014).
8. Harvey, C. M., Wood, J. D., & Wang, S. Brittle interfacial cracking between two dissimilar elastic layers: Part 1—Analytical development. *Compos. Struct.* **134,** 1076–1086 (2015).
9. Harvey, C. M., Wood, J. D., & Wang, S. Brittle interfacial cracking between two dissimilar elastic layers: Part 2—Numerical verification. *Compos. Struct.* **134,** 1087–1094 (2015).
10. Wood, J. D., Harvey, C. M. & Wang, S. Partition of mixed-mode fractures in 2D elastic orthotropic laminated beams under general loading. *Compos. Struct.* **149,** 239–246 (2016).
11. Wood, J. D., Harvey, C. M. & Wang, S. Effect of Poisson's ratio mismatch on brittle

interfacial cracking between two dissimilar elastic layers. *Compos. Struct.* **158,** 56–63 (2016).

12. Harvey, C. M., & Wang, S. Experimental assessment of mixed-mode partition theories. *Compos. Struct.* **94,** 2057–2067 (2012).

13. Harvey, C. M., Eplett, M. R. & Wang, S. Experimental assessment of mixed-mode partition theories for generally laminated composite beams. *Compos. Struct.* **124,** 10–18 (2015).

14. Wang, S., Harvey, C. M. & Wang, B. Room temperature spallation of α-alumina films grown by oxidation. *Eng. Fract. Mech.* **178,** 401–415 (2017).

15. Harvey, C. M., Wang, B. & Wang, S. Spallation of thin films driven by pockets of energy concentration. *Theor. Appl. Fract. Mech.* **92,** 1–12 (2017).

16. Yuan, B., Harvey, C. M., Thomson, R. C., Critchlow, G. W. & Wang, S. Telephone cord blisters of thin films driven by pockets of energy concentration. In review (2017).

17. Harvey, C. M., Wang, S., Yuan, B., Thomson, R. C. & Critchlow, G. Determination of mode I and II adhesion toughness of thin films by circular blister tests. In review (2017).

18. Sun, C. T. & Jih, C. J. On strain energy release rates for interfacial cracks in bi-material media. *Eng. Fract. Mech.* **28,** 13–20 (1987).

19. Koenig, S. P., Boddeti, N. G., Dunn, M. L. & Bunch, J. S. Ultrastrong adhesion of graphene membranes. *Nat. Nanotechnol.* **6,** 543–546 (2011).

20. Tolpygo, V. K. & Clarke, D. R. Spalling failure of α-alumina films grown by oxidation: I. Dependence on cooling rate and metal thickness. *Mater. Sci. Eng. A* **278,** 142–150 (2000).

21. Tolpygo, V. K. & Clarke, D. R. Spalling failure of α-alumina films grown by oxidation. II. Decohesion nucleation and growth. *Mater. Sci. Eng. A* **278,** 151–161 (2000).

22. Ni, Y., Yu, S., He, L. & Jiang, H. The shape of telephone cord blisters. *Nat. Commun.* **8,** 1–6 (2017).

23. Faou, J. Y., Parry, G., Grachev, S. & Barthel, E. How does adhesion induce the formation of telephone cord buckles? *Phys. Rev. Lett.* **108,** 1–5 (2012).

24. Cordill, M. J., Bahr, D. F., Moody, N. R. & Gerberich, W. W. Adhesion measurements using telephone cord buckles. *Mater. Sci. Eng. A* **443,** 150–155 (2007).

25. Jensen, H. M. The blister test for interface toughness measurement. *Eng. Fract. Mech.*

40, 475–486 (1991).

26. Jensen, H. M. Analysis of mode mixity in blister tests. *Int. J. Fracture* **94,** 79–88 (1998).

27. Cao, Z., Tao, L., Akinwande, D., Huang, R., & Liechti, K. M. Mixed-mode traction-separation relations between graphene and copper by blister tests. *Int. J. Solids. Struct.* **84,** 147–159 (2016).

28. Williams, J. G. Energy release rates for the peeling of flexible membranes and the analysis of blister tests. *Int. J. Fracture* **87,** 265–288 (1997).

29. Hencky, H. Über den spannungszustand in kreisrunden platten mit verschwindender biegungssteiflgkeit. *Z. für Mathematik und Physik* **63,** 311–317 (1915).

30. Wang, Y. & Tong, L. Closed-form formulas for adhesion energy of blister tests under pressure and point load. *J. Adhesion* **92,** 171–193 (2016).

31. Kitt, A. L. et al. How graphene slides: Measurement and theory of strain-dependent frictional forces between graphene and SiO_2. *Nano Lett.* **13,** 2605–2610 (2013).
